# Ground Glass-Like Inclusions: Associated with Liver Toxicity

**DOI:** 10.5146/tjpath.2020.01510

**Published:** 2021-01-15

**Authors:** Kemal Deniz

**Affiliations:** Department of Pathology, Erciyes University, Faculty of Medicine, Kayseri, Turkey

**Keywords:** Pseudoground glass, Ground glass, Toxicity, Herbal, Mushroom, Liver

## Abstract

***Objective:*** The etiology of ground glass-like inclusions is heterogenous and the pathology has been described in various conditions including HBV infection, Lafora’s disease, fibrinogen storage disease, type IV glycogenosis, and alcohol reversion therapy. Similar ground glass-like inclusions are also associated with immunosuppressed conditions and multiple medications, for which the clinical significance is still unclear. Additional cases, some with previously unreported unique etiologies, and their follow-up were described in this study.

***Materials and Methods: *** Eleven cases were examined between 2008 and 2019 for this study. The clinical data and histologic slides were reviewed. All of the cases were negative for Hepatitis B virus. None of the patients declared alcohol intake or a history of epilepsy.

***Results: *** Liver histology showed mild lobular inflammation in most of the cases (72%). Ground glass-like hepatocytes were distributed in the patchy-panlobular, periportal, and centrizonal pattern at 55%, 27%, and 18%, respectively. Clinical history revealed medication use in nine (82%) patients including NSAIDs, steroids, and chemotherapy. Ground glass-like inclusions were related to herbal toxicity in two of the patients. Liver function tests were elevated in all of the cases. Follow-up data revealed four patients with malignancy who died of their cancer. Seven patients showed resolution of elevated liver enzymes with a median follow-up period of 37 months (range 7-132 months).

***Conclusions: *** Medication is the most relevant etiology for the development of these inclusions. Ground glass-like inclusions may also seen in herbal toxicity. Transplantation was not an etiologic factor in our patients. Most of the patients displayed an indolent course with resolution of the elevated transaminases.

## INTRODUCTION

Cytoplasmic ground glass appearance of the liver cells was described in patients with Hepatitis B infection by Hadziyannis et al. in 1973 (1). Similar-appearing inclusions were then later described in a variety of etiologies including myoclonic epilepsy, Type IV glycogenosis, alcohol reversion therapy, and fibrinogen storage disease (2). In 1985, three bone marrow transplant patients with bone marrow who had eosinophilic inclusions in hepatocytes were reported (3). These inclusions were named as pseudoground glass change by Wisell et al. in 2006. In the current paper, 11 new cases, some with new apparent etiologies, were described together with the natural history, and a review of the previously reported cases was additionally provided (4).

## MATERIAL and METHODS

The files of the Pathology Department between 2008 and 2019 were searched for “pseudoground glass hepatocytes” and “ground glass-like hepatocytes”. Eleven cases were included in this study. Ethical approval was obtained from the Institutional Clinical Research Ethics Committee (approval number 2020/241). The clinical data and medical history of the patients were reviewed. None of the patients declared alcohol intake or a history of epilepsy.

The slides of the liver needle biopsies were evaluated for the degree and distribution of ground glass-like hepatocytes, steatosis, inflammation, and fibrosis. Immunohistochemical staining for HBSAg (1:50 dilution; Thermo Fisher Scientific, Fremont, CA) was performed by using the Ventana Bench Mark autostainer. Masson’s trichrome and PAS stains were performed on the Ventana Bench Mark special stains system. The phosphotungstic acid-hematoxylin stain was also performed.

## RESULTS

**Clinical Features: **Seven of the 11 patients were male and 4 were female (M:F=1.75). Their ages ranged between 17 and 75 years with a median of 37 years. Four patients had an underlying malignancy including lymphoma, acute myeloblastic leukemia, and gastric or lung cancer. One patient had chronic obstructive lung disease and one had hyperthyroidism. An underlying disease was not found in 4 of the 11 patients. The clinical history revealed medication use in nine (82%) patients. Two patients were undergoing chemotherapy (FOLFIRI and R-CHOP), three patients were using NSAIDs, and two were using steroids. A detailed drug list is given in Table 1. Patient 2 declared that she was using home-made stinging nettle extract twice daily for weight loss. The medical history of patient 11 revealed consumption of wild oyster mushrooms.

**Table 1:** Clinicopathologic features of patients with ground glass-like hepatocytes.

**Patient**	**Age/ Gender**	**Clinical history**	**Medication**
Patient 1	61/M	Chronic obstructive lung disease	Steroid
Patient 2	26/F	None	Stinging nettle extract
Patient 3	75/M	Large B cell lymphoma	R-CHOP+Rituximab
Patient 4	57/M	Lung carcinoma, diabetes	NSAID, Tramadol
Patient 5	37/M	Hyperthyroidism	NSAID, Methimazole
Patient 6	33/F	Acute myeloblastic leukemia	Valtrex, steroid
Patient 7	36/M	None	NSAID
Patient 8	17/M	None	Doxycycline
Patient 9	66/F	Osteoporosis	Zolendronate, Teriparatide
Patient 10	33/F	Gastric carcinoma	5-FU, Irinotecan
Patient 11	56/M	None	Mushroom

Viral hepatitis serology, including hepatitis A, B, C, and E, and autoimmune serology for ANA, ASMA, anti-LKM1, AMAM2, anti-SLA, were negative. All of the cases had elevated ALT and AST levels with a range of 107-754 U/L and 41-693 U/L respectively. ALP levels were elevated in 3 of the 11 patients within a range of 179-1762 U/L. The bilirubin level was mildly increased in one of these patients.

Follow-up data revealed that four patients died of their malignant disease. The rest of the seven patients were followed-up during a median period of 37 months (range 7-132 months). Patients showed resolution of elevated liver enzymes within a median period of 50 days ranging from 26 to 150 days. Six of the seven patients were free of liver disease, and control liver enzymes were within normal limits. Only patient 8 showed a second peak of liver enzyme elevation during a follow-up period of 33 months.

**Figure 1:**
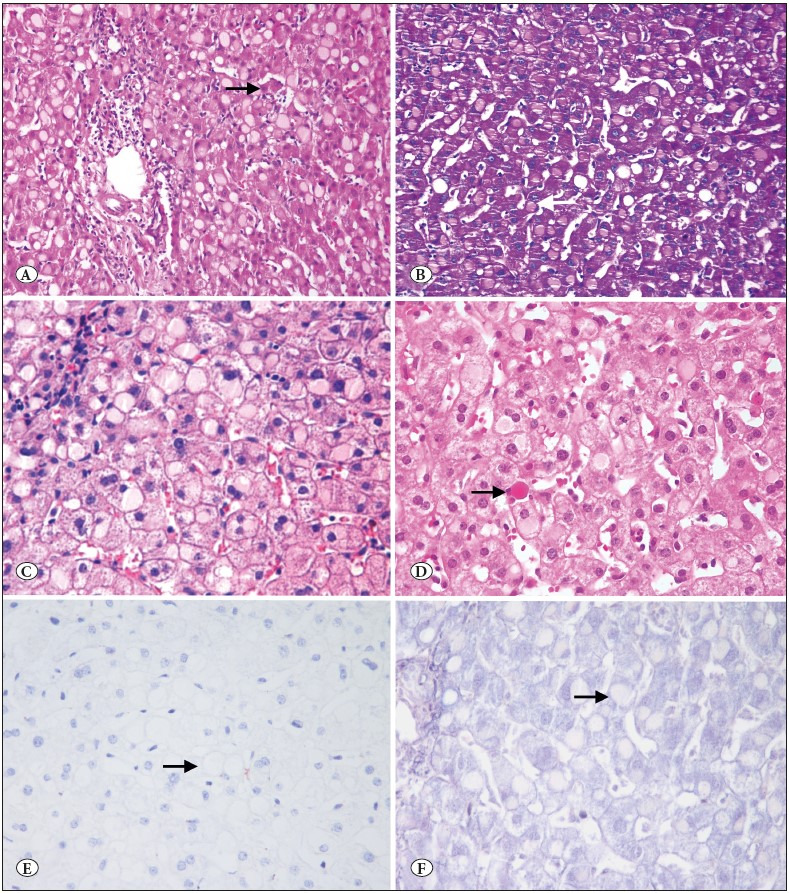
**A)** Liver biopsy showing pale eosinophilic, round ground glass-like inclusions in the periportal hepatocytes. Mild lobular (arrow) and portal inflammation was noted (H&E; x200). **B)** PAS positivity in ground glass-like inclusions (PAS; x200). **C)** At higher magnification, pale eosinophilic ground glass-like inclusions in the hepatocyte cytoplasm (H&E; x400). **D)** Ground glass-like inclusions and scattered apoptotic hepatocytes (arrow) (H&E; x400). **E)** Ground glass-like inclusions are negative for HBsAg immunostain (arrow) (IHC; x400) and **F)** Phosphotungstic acid-hematoxylin stain (arrow) (PTAH; x400).

**Histologic Features: **Histologic evaluation showed round eosinophilic inclusions, surrounded by a thin rim of hepatocyte cytoplasm. Some of them had a clear halo (possibly a retraction artifact) between the inclusion and cytoplasm. The nuclei were usually located at the periphery of the cell (Figure 1). These inclusions were positively stained with PAS (Figure 1). HBsAg immunostains were all negative (Figure 1). Phosphotungstic acid-hematoxylin stain for fibrinogen inclusions was negative in all cases (Figure 1). Ground glass-like hepatocytes were distributed in a patchy-panlobular pattern in 6 (55%) of the 11 patients. The rest of the patients (45%) showed a zonal distribution, including 3 cases with periportal and 2 cases with centrizonal pattern. The most frequent histologic changes associated with ground glass-like hepatocytes were mild lymphocytic lobular inflammation with scattered acidophil bodies (Figure 1) and focal necrosis. Lobular inflammation was found in 72% of the patients. Confluent necrosis and bridging necrosis were not seen in any case. Two cases (18%) showed mild zone 3 cholestasis and mild ductular reaction. Mild portal lymphocytic infiltrate and mild periportal fibrosis were present in one patient (9%). Mild steatosis was present in one patient (9%). Histologic features of steatohepatitis including ballooning and pericellular (“chicken-wire”) fibrosis were not observed.

## DISCUSSION

Adaptive changes in hepatocytes similar to ground glass hepatocytes have been described in patients with anticonvulsant drugs, rifampicin, cholorpromazine, and multi-drug use (5). Zubair et al. reported three patients with PAS+ ground glass-like hepatocytes in an autopsy study of bone marrow transplant patients. All patients were treated with cyclophosphamide and methotrexate (3). Ground glass-like change was also reported in a patient treated with mycophenolate, prednisone, and azothiopirine for autoimmune hepatitis (6). Inclusions were reported to be associated with hepatic outflow occlusion in another patient (7). Two other case series of ground glass-like change were reported in 2006 (4,8). Lefkowitch et al. reviewed 10 patients with similar inclusions and they found that seven of their patients were transplant recipients (5 with hematopoetic stem cell transplant, 2 with liver transplant). All patients had a history of multidrug use lasting several months. Immunosuppressive drugs including mycophenolate, tacrolimus, and steroids were commonly used by these patients. Graft versus host disease (4 patients), acute cellular rejection (2 patients), chronic hepatitis (1 patient), and veno-occlusive disease (1 patient) were associated with ground glass-like hepatocytes on histologic examination of the liver biopsies (8). Wisell et al. found that nine of the 12 patients were related to immunosuppression, including transplantation, HIV infection, renal dialysis and inflammatory bowel disease. All of the 12 patients were using polypharmacotherapy, mostly including steroids and tacrolimus (FK506)(4). Similarly, all three patients in the series by Bejarano et al. were undergoing multidrug medication including steroids and tacrolimus (9).

In summary of the above studies, PAS+ ground glass inclusions have been mostly reported in immunosuppressed patients on multidrug medications in a setting of transplantation and immunosuppression. Transplantation was not an etiologic factor in our patients. Also, in contrast to previously published data, an underlying disease was not found in 4 of our 11 patients. Clinical history revealed medication use in nine (82%) patients including NSAIDs, steroids, and chemotherapy. Stinging nettle extract and wild oyster mushroom consumption were the possible causative factors for PAS+ ground glass hepatocytes in two patients, which is an unreported finding in English literature. Given this finding, one might speculate that herbal preparations and other alternative medicines may also lead to toxic effects that may mimic all kind of injury on liver biopsy, including ground glass change. Thus, herbal and mushroom toxicity with ground glass change add new data about the histologic spectrum of this entity.

There were no consistent liver function test results in the literature review. A total of 25 patients (52%) from three reported case series showed only mildly elevated enzymes (4,8,9). All of the cases in our series were found to have elevated ALT and AST levels. However, ALP levels were also elevated in 3 of the 11 patients. Follow-up data revealed four patients in this series who died of malignant disease. The rest of the seven were followed-up during a median period of 37 months (range 7-132 months). Patients showed resolution of elevated liver enzymes in a median period of 50 days, ranging between 26-150 days. Most of our patients were free of liver disease, and control hepatic enzymes were within normal limits. Only one patient showed a second peak of liver enzyme elevation during a follow-up period of 33 months. The clinical significance of ground glass-like hepatocytes is unclear; however, our patients without malignant disease displayed an indolent course, not associated with severe liver disease. Thus it seems that abnormal liver test results were mostly related to the accompanying disorder rather than ground glass-like inclusions.

The histological distribution of the ground glass-like inclusions in previous reports as well as our study varies from case to case. Panlobular-patchy distribution was the most frequent pattern in our series similar to Wisell et al. (4). However, periportal predilection was also reported (8). Other histological features of the liver biopsies included mild lobular inflammation in most of our patients. Severe acute hepatitis and chronic hepatitis with fibrosis were not compatible with ground glass-like change. Mild cholestasis and mild steatosis were seen in a minority of the cases.

The pathogenetic mechanism of PAS+ ground glass-like hepatocytes related to immunosuppression as well as medications and other alternative herbal or medicinal agents remains unclear. It is hypothesized that ground glass change in these settings is a result of glycogen accumulation due to enzymatic inhibition in glucose metabolism (4). Ground glass-like inclusion may thus reflect subcellular hepatocyte injury with abnormal glycogen deposition (10). Electron microscopy revealed glycogen accumulation with degenerated organelles. Glycogen inclusions are nonmembrane bound aggregates of beta glycogen granules with endoplasmic reticulum. The inclusions resemble polyglucosan bodies, which are considered ‘unbranched equivalent of glycogen’ as possibly seen in GSD4. It remains unclear whether it is an adaptive response to the induction of hepatocytes by the medication (8,9,11).

Most of our patients with ground glass-like change displayed an indolent course. A literature review and our own series showed that the most likely explanation for the development of ground glass-like hepatocytes is the medication/drug effect. Transplantation was not a causative factor in this study, in contrast to previous data. Herbal liver toxicity may also cause hepatocyte changes similar to ground glass morphology.

## CONFLICT of INTEREST

The author declares no conflict of interest.

## FUNDING

None
